# Annotations

**Published:** 1889-01-26

**Authors:** 


### ANNOTATIONS.

**Smoking.** THE subject of smoking has given rise to perennial controversies. It does not appear to have occurred to opposed reasoners at all times that the varying effects produced by the use of tobacco are to be explained by the constitutional peculiarities of those who use it. Dr. W. S. Porter, of Sheffield, has been saying a few words in favour of the weed, and has apparently brought down upon his head the rebuke of another doctor who takes an opposite view. But Dr. Porter's temper and reasoning will surely commend themselves to all men who have attained an age when reason may be supposed to have become stronger than mere passion or fanaticism. "All I contended for," says Dr. Porter, "I still maintain, that moderate smoking by adults, tolerant of tobacco, has not been shown to be hurtful, and may under some circumstances even be beneficial. I do not deny for a moment that excessive smoking may be followed by very injurious consequences; but I believe that in proportion to the popularity of the practice, such results are comparatively seldom met with by medical men." The temperateness and reasonableness of this language is worthy of the judicial bench. It puts the whole question in a nutshell, and really says the wisest possible word on the subject. The present writer has been a smoker and a non-smoker; a smoker in small and a smoker in large quantities. He has for some years given up the practice of habitual smoking. His conclusions, both from his personal and professional experience, are identical with those of Dr. Porter. Tobacco in excess may, and often does, produce bad results. Young men should smoke little, if at all; but adults, tolerant of tobacco, may generally smoke in moderation, not only without injury, but sometimes with advantage. Dr. Porter would probably agree with this proposition, that on the whole the habit of smoking probably does more good than harm. Be that as it may, there is no doubt that fanaticism of every kind is injurious; that extreme opinions are usually extremely wrong; and that generally the middle course is the safest.

**Cremation.** SPEAKING at Leeds the other day, Dr. Jayne, Bishop-Designate of Chester, said "the old principle of 'ashes to ashes and dust to dust' was prevented from being carried out by our present method of burial. Science, religion, art, and economy, all said the present system was monstrous; but patience was required to educate public opinion in the matter." It is well that public opinion should not be too flexible and mobile; but it is also well that it should not be too obstinate and unyielding. Where there is little sentiment, there will be great flexibility of opinion; where the sentiment is deep and strong, opinion will be almost immovable. Hence the hard death of old superstitions of every kind, and hence also the slow progress of many changes which reason and necessity alike insist upon demanding. Cremation is a mode of burial which commends itself to every intelligent sanitarian. It is practically certain that if we were not accustomed to the interment of dead bodies in the ground, we should regard with horror a new suggestion to dispose of them in that way. If we could imagine ourselves the first people in the world, without any experience of death, it is a question well worth considering what would be the mode of disposing of the dead which would suggest itself to our inexperience? The probability is that we should not want to dispose of them at all. Those at any rate whom we had deeply loved, we should desire to keep always with us; and if there were no such thing as decomposition, that natural feeling would be very generally indulged. But if in our inexperience we should keep the dead by us, we should soon be compelled by the horrors of their advancing putridity to put them away, not only out of sight, but also beyond the ken of all the other senses. It is very likely that in this way interment in the ground originally began, and finally established itself as the almost universal form of burial. It was a way of disposing of the dead which the living themselves were compelled to choose, for the sake of their own well-being. There lies the gist of the whole question. Does that method now subserve the purpose it formerly served? That is our problem in these later days. It certainly does not. We have to ask ourselves as practical people exactly the question our remotest ancestors asked themselves: How can we best dispose of our dead so as to show reverence for them and regard for the living? Our progenitors found burial in the ground the most convenient, most economical, and most effectual way of disposing of their dead. In our larger cities at the present day it is neither convenient, economical, nor effectual. On the contrary, its inconvenience is notorious, and particularly in London, where many miles must often be travelled, and hours of slow agony endured in performing the last rites of sepulture. It is not economical, but shamefully and sometimes ruinously extravagant. It is not effectual, because on all hands we now find both the air and the water used by the living poisoned by the decaying dead. We should consider all these things in the light of reason; and try to imagine ourselves for the first time facing the problem of the disposal of our lost ones. If we were to do that, there is no doubt that a vast majority would infinitely prefer the rapid return of "dust to dust" by fire to the slow horrors of decomposition and putridity in the ground.

**Coaxing the Appetite.** A GOOD many people do not know what it is to have a keen relish for food. Women, whose lives are mostly spent within the four walls of their dwellings, are often very dainty and feeble in their table performances. Yet food is the parent of health, comfort, and efficiency. Without sufficient food neither man nor woman can be happy or well. When a physiologist has made an announcement of that kind, he has not merely uttered a scientific dictum; he has also stated a moral proposition. Is it the duty of every man and woman to be and to do his best in life's struggle? If every one answers this in the affirmative, then this affirmation must also be agreed with: If it be every man's duty to be and to do his best, it is every man's duty to employ the means that will enable him to be and to do his best. Therefore, also, it is everybody's duty to eat sufficient food. Many women have poor appetites. The reason for this is not merely that they live indoors, though it is mainly that; but the incessant small worries, the culinary cares, the thousand calls upon a woman's attention—these all tend to dull the edge of appetite, and to make eating rather a bore and a nuisance than a pleasure. Eating ought to be a pleasure; but if it cannot be made a pleasure, it both can and ought to be made a duty. Most Englishwomen believe in duty, and it is to them we make this short but not unimportant appeal. The appetite can be coaxed and trained as well as any other part of the body. The comfortable wearing of clothes is the result of training. What a long and wearisome training and coaxing the fingers require before they will play the piano or the violin with ease and pleasure! Everyone who has been accustomed to riding and driving without gloves in early boyhood knows how exceedingly uncomfortable it is to have to handle reins and whip when manhood demands that the hands shall be encased in thick leather. The genuine savage hates clothing and gloves, just as the ill-disciplined woman who has no appetite hates the duty of eating. But there is no denying this fact, that the woman who, not being ill, refuses to take reasonable meals as a matter of duty, is on precisely the same moral and intellectual level as the savage who refuses to be worried with the discomfort of clothes. There is no danger in eating a fair quantity of food three or four times a-day, even though the appetite be wanting and digestion be not good. If both digestion and appetite are to be improved, two important rules must be observed. The first is, that really appetising food must be prepared three or four times daily; the second, that a fair quantity of this must be eaten each time. Of course, there may result for a period some of the symptoms of uncomfortable dyspepsia; but remedies may be obtained for these, or they may be endured. The one thing to bear in mind is this: that perseverance in eating will at length produce an appetite, and will bring health, pleasure, and efficiency in its train.

